# Insulin in the regulation of the renin-angiotensin system: a new perspective on the mechanism of insulin resistance and diabetic complications

**DOI:** 10.3389/fendo.2024.1293221

**Published:** 2024-01-23

**Authors:** Tatyana S. Zamolodchikova, Svetlana M. Tolpygo, Alexander V. Kotov

**Affiliations:** Physiology of Motivation Laboratory, P. K. Anokhin Institute of Normal Physiology, Moscow, Russia

**Keywords:** insulin, renin-angiotensin system, insulin resistance, signaling cross-talk, angiotensin, diabetes mellitus

## Introduction

Insulin, a polypeptide hormone that is produced in the β-cells of the pancreatic islets of Langerhans, has multifaceted effects on metabolism in virtually all tissues. Insulin facilitates glucose entry into cells, stimulates glycogen formation in liver and muscle, and enhances fat and protein synthesis (1). Insulin also has a mitogenic function, stimulating cell growth and proliferation (2). By crossing the blood-brain barrier, insulin can affect feeding and cognition through CNS mechanisms (3). Binding of insulin to its receptor tyrosine kinase (RTK) triggers signal transduction, with phosphorylation of cellular substrates (IRS) and activation of phosphatidylinositol-3-kinase (PI3K), which initiates a chain of events directly involved in the metabolic and mitogenic effects of insulin (1, 4). The second pathway involves activation of mitogen-activated protein kinases (MAPK), which play a predominant role in the control of mitogenic effects of insulin (5). Disruption of the IRS/PI3K pathway leads to decreased tissue sensitivity to the metabolic action of insulin - a state of insulin resistance (IR), which is characteristic of patients with type 2 diabetes (T2D), obesity and arterial hypertension (6, 7).

The renin-angiotensin system (RAS) contributes to the pathophysiology of IR – thus angiotensin II (ANGII) disrupts insulin signaling by promoting phosphorylation of the insulin receptor and IRS-1 and PI3K which impairs their function (8–11). Given the close relationship between these signaling systems, we hypothesize that insulin itself may influence the RAS and regulate its function. We also discuss possible pathological consequences of RAS dysfunction due to impaired insulin signaling relevant to IR and diabetes complications.

## Briefly about RAS - components and function

As a universal regulatory system, the RAS ensures normal functioning of basic life processes such as hemodynamics, fluid-salt and glucose homeostasis, behavior, and also participates in the regulation of immunity, apoptosis, inflammation and many others (10, 12–14). The major peptide effector of RAS, ANGII, is formed from the protein precursor angiotensinogen (AGT) by proteolytic processing involving renin and angiotensin-converting enzyme (ACE) (12). ANGII acts through two subtypes of G-protein-coupled receptors (GPCRs), the AT1R and AT2R receptors. Alternative processing of AGT involving ACE2 results in the formation of other angiotensins - ANG-(1-7) and ANGIV, which have their own receptors, Mas-receptor and IRAP (insulin-regulated aminopeptidase), respectively (12, 14–17).

Physiological effects of RAS are achieved through the interaction of counterbalancing activity pathways: “pathophysiological” ACE/ANGII/AT1R, whose activation is associated with hypertension, oxidative stress, hypertrophy, fibrosis and inflammation, and “protective” ACE/ANGII/AT2R, which has the opposite function, including hypotensive and anti-inflammatory effects (18). Alternative pathways involving ANG-(1-7) and AngIV in general function within the “protective” RAS axis (15–17).

In addition to the central RAS circulating in the blood system, there are local RAS in the brain, pancreas, kidneys, intestines, etc. involved in the regulation of the functional activity of these organs (18–21). Thus, pancreatic RAS, represented by a full set of functional components, including ANGII and ANG-(1-7) and their receptors, affects cell proliferation, apoptosis, oxidative stress, and inflammation (21–23). It has been shown that islet RAS regulates insulin biosynthesis and secretion, affects the mass of islet β−cells and is able to alter glucose homeostasis (24).

## Evidence of the effect of insulin on the activity of RAS components

**Figure 1:**
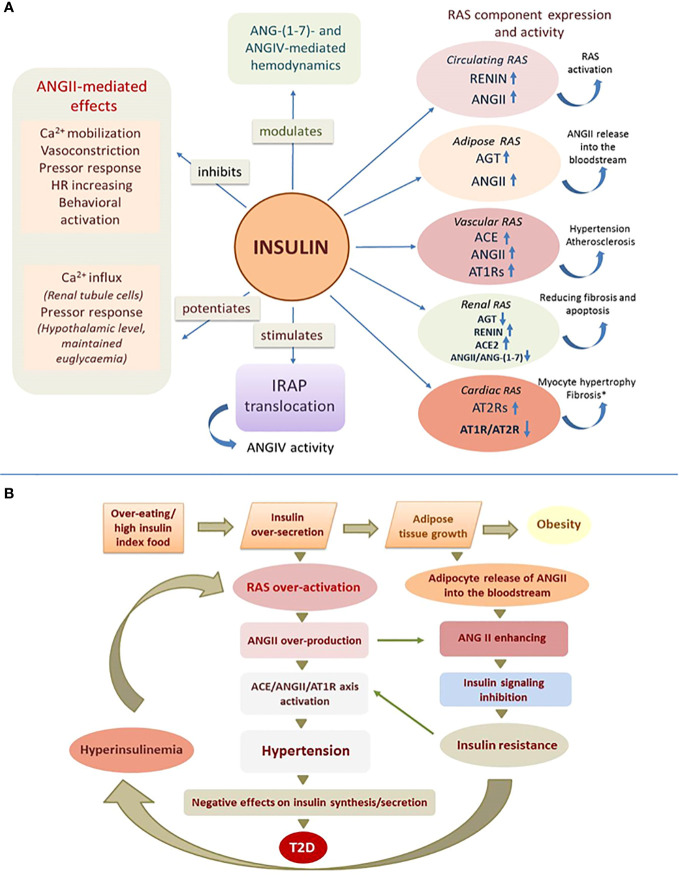
Effect of insulin on RAS function. **(A)** Insulin affects central and local RAS by acting on their receptors, enzymes and angiotensin peptides (experimentally confirmed effects, details in the text of the article); ANG, angiotensin; AGT, angiotensinogen; ACE, angiotensin-converting enzyme; AT1R and AT2R, angiotensin receptors; IRAP, insulin-regulated aminopeptidase; (↑) – increasing; (↓) – reducing; (*) - high concentrations of insulin. **(B)** Mechanism of angiotensin-dependent IR as a vicious circle.

Insulin has a significant effect on RAS function by affecting the expression, secretion, and activity of its components (**Table 1**, **Figure 1A**).

**Table 1:** Effect of insulin on the renin-angiotensin system (summary of cited literature).

Effect of insulin on RAS	Model	Experimental details	Ref.
Secretion and activity of RAS components			
Increase in plasma renin and ANGII activity	Humans (healthy male volunteers; 26-27 years)	Euglycemic insulin clamp (160 μU/ml)	(25)
Increase in AGT protein and ANGII secretion	Human abdominal subcutaneous adipocytes (from female subjects; 40 -50 years; tissue culture)	Treatment with insulin (1-1000x10^-9^ M; 48 h)	(26)
Increase in renin secretion in the renal cortex	Renal cortical slices (rats, Sprague-Dawley normal males; 180-220 g)	Treatment with insulin (3.5x10^-9^ M; 30 min)	(27)
Expression of renal RAS components			
Suppression of AGT gene expression	Rat immortalized renal proximal tubular cells (cell culture)	Incubation with insulin (10^-7^ M; 24 h)	(28)
Increase in ACE2 gene expression	Mouse growth-restricted, conditionally immortalized podocytes (cell culture)	Incubation with insulin (2x10^-7^ M; 1, 24, 48 h)	(29)
Expression of vascular and cardiac RAS components			
Suppression of AGT and renin mRNA expression Stimulation of ACE activity (high doses of insulin)	Human aortic endothelial cells (cell culture)	Incubation with insulin (10, 100, 1000 μU/ml; 48 h)	(30)
Overexpression of AT1_a_R receptors	Rat aortic VSMCs (cell culture)	Incubation with insulin (10^-7^ M, 24 h)	(31)
Decrease in AT1_a_R expression; Increase in AT2R expression; Decrease in AT1_a_R/AT2R ratio	Tissue sections of rat ventricle (Sprague-Dawley, normal females (230 ± 8 g)	Chronic hyperinsulinemia (insulin osmotic minipumps 2 U/day; 1 μl/h; 7 wk)	(32)
Modulation of angiotensin peptide action			
Reduce in ANGII–induced Ca^2+^ response (insulin sensitive subjects)	Human skin fibroblasts (from hypertensive (age 50 ± 8 years) insulin-sensitive and insulin-resistant males; cell culture)	Preincubation with insulin (10^-7^ M; 20 min)	(33)
Decrease in ANGII-induced Ca^2+^ response and cell contraction	Rat mesangial cells (Wistar males; cell culture)	Preincubation with insulin (5 μg/ml; 2 h)	(34)
Reduce in ANGII-induced Ca^2+^ response	Rat VSMCs (Wistar-Kyoto males; cell culture)	Preincubation with insulin (0.5x 10^-9^ M; 10 min)	(35)
Inhibition of ANGII -induced cell contractions Decrease in ANGII –induced Ca^2+^ response	Dog VSMCs (cell culture)	Preincubation with insulin (40 μU/mL; 20 min or 7 day)	(36)
Decrease in ANGII -induced Ca^2+^ response	Rat aortic VSMCs (Sprague-Dawley, normal males, 250-300g; cell culture)	Preincubation with insulin (10 μU/ml - 100 mU/ml; 20 min)	(37)
Stimulation of the Ca^2+^ influx induced by ANGII	Isolated rat cortical thick ascending limb (Sprague Dawley, normal males; 130 –180 g)	Preincubation with insulin (10^-7^ M; 20 min)	(38)
Reduction of ANG II-induced arterial and venous contraction	Isolated femoral artery and vein (rabbits, males; 2.5-3.5 kg)	Incubation with insulin (0.12-120 mU/ml)	(39)
Reduction of hemodynamic and behavioral effects of ANGII	Rats (Wistar, normal males; 350-400 g)	Pretreatment with insulin (1 U intraperitoneally; 30 min)	(40)
Reduce in the pressor response to ANGII Increase in the pressor response to ANGII (sustained euglycemia)	Rats (Wistar, normal males; 350-400 g)	Continuous infusion of insulin (i.v.; 60 pmol kg^-1^min^-1^) Sustained euglycemia (insulin 2-600 pmol kg^-1^ min^-1^ +glucose 5 – 15%)	(41)
Increase in the pressor response to ANGII	Rats (Sprague-Dawley, normal males; 250-300 g)	Insulin perfusion (i.c.v., 12 mU/h; 2 h at a flow rate of 4 μl/h)	(42)
Modulation of hemodynamic effects of ANG-(1-7)	Rats (Wistar, normal males; 350-400 g)	Pretreatment with insulin (1 U intraperitoneally; 30 min)	(43)
Modulation of hemodynamic effects of ANGIV	Rats (Wistar normal males; 350-400 g)	Pretreatment with insulin (1 U intraperitoneally; 30 min)	(44)

ANG, angiotensin; AGT, angiotensinogen; ACE, angiotensin-converting enzyme; AT1R and AT2R, angiotensin receptors.

### Insulin promotes an increase in circulating ANGII levels

Insulin increases circulating ANGII levels by stimulating plasma renin activity (25) and also increases AGT expression and ANGII secretion in subcutaneous adipose tissue, which is an important source of circulating ANGII (26).

### Insulin affects the expression of components of local RASs

In renal RAS, insulin stimulates renin production (renal cortex) (27), and suppresses AGT expression by interacting with a specific insulin-responsive element located in the promoter region of the AGT gene (proximal tubule cells) (28). In cultured podocytes, insulin increases the expression of ACE2, responsible for the production of ANG-(1-7), a functional ANGII antagonist, which shifts the ANGII/ANG-(1-7) ratio in favor of an “anti-ANG II” profile and contributes to reduced fibrosis and apoptosis (29).

In the vascular RAS (aortic endothelial cells) insulin suppresses AGT and renin expression but stimulates ACE biosynthesis (30). Insulin also induces AT1R overexpression in blood vessels, which may lead to increased biological efficacy of ANGII and thereby induce hypertension and atherosclerosis (31). In cardiac tissue, high concentrations of insulin cause the opposite effect, altering the AT1R/AT2R ratio in favor of an increase in AT2R, leading to pathological changes in the organ (32).

### Insulin modulates the action of angiotensin peptides

Insulin reduces ANGII-induced Ca^2+^ response in various cell types such as human skin fibroblasts and mesangial cells (33, 34) as well as in vascular smooth muscle cells (VSMCs) of various origins (34–37). In rat renal tissue (cortical thick ascending limb), insulin does not alter ANGII-induced Ca^2+^ release from intracellular reservoirs but stimulates Ca^2+^ influx (38). Insulin dose-dependently suppresses the vasoconstrictive effects of ANGII in arterial and venous systems, suggesting that insulin may modulate ANGII-mediated vascular function (39).

Intraperitoneal administration of 1 U of insulin abolishes ANGII-mediated activation of complex instrumental behavior in rats and halves the hypertensive effect of ANGII (40). Continuous insulin infusion (i.v.) reduces the pressor response to ANGII in rats and, conversely, increases it if euglycemia is artificially maintained (41). Centrally administered (i.c.v.) insulin potentiates ANGII pressor effects at the hypothalamic level (42).

Insulin regulates the activity of ANGII bioactive derivatives, ANG-(1-7) and ANGIV, which elicit responses generally opposite to ANGII. When rats were injected intraperitoneally with 1 U of insulin, the weak hypotensive effect of ANG-(1-7) changed into a biphasic hyper-hypotensive effect (43). In a similar experiment, the weak hypotensive effect of ANGIV on the background of insulin was changed to hypertensive (increase in blood pressure by 11-16%) with subsequent prolonged vasodilatation and tachyarrhythmia (44).

### Insulin regulates IRAP function

The specific ANGIV receptor, IRAP, is an insulin-regulated aminopeptidase with which ANGIV interacts as an inhibitor (45). Expressed in the brain, heart, kidney, and blood vessels, IRAP is involved in the regulation of blood flow, glucose metabolism, and processes related to cognition, as well as several other functions (46). Insulin stimulates the translocation of IRAP together with the insulin-responsive glucose transporter GLUT4 from intracellular vesicles to the cell membrane, where IRAP performs its function by cleaving vasopressin, somatostatin and other active peptides (47).

## Discussion

The study of the functional relationship between insulin and renin-angiotensin signaling systems, which play a key role in the control metabolism and homeostasis, seems particularly important due to the confirmed clinical association between IR and hypertension (48). Insulin signaling system and RAS are in a balanced relationship of reciprocal regulation, where the activity of each system depends to a certain extent on the functioning of the other. Thus, ANGII regulates insulin secretion by activating AT1Rs on the surface of β−cells (24) and plays an important role in the development of IR, which occurs in various pathological conditions, including diabetic (8–10). Insulin, in turn, affects central and local RASs by acting on their receptors, enzymes, and angiotensin effector peptides (**Figure 1A**).

We suggest that RAS activity is largely dependent on the state of the insulin signaling system and this may be extremely important for both normal body functioning and the development of pathology. This regulation can be flexible and efficient under physiological conditions and dramatically impaired under pathological conditions. Normally, insulin can increase ANGII concentrations and at the same time counterbalance the hyperactivity of this hormone by suppressing its effects, causing a kind of temporary “insulin-dependent resistance to ANGII”. Possible mechanisms for the inhibitory effect of insulin on ANGII signaling in this case could be chemical modification and inactivation of angiotensin receptors, as insulin-activated RTKs are able to phosphorylate GPCRs (49) or “borrow” components of GPCR signaling, including β-arrestins and G-protein-coupled kinases (50). In the absence of the hypoglycemic effect of insulin, which is characteristic of the IR state, insulin may, in contrast, enhance the hypertensive properties of ANGII (41) and thereby contribute to the hypertension that usually accompanies IR.

RAS hyperactivation is characteristic of the IR state, but in this case a chicken-or-egg situation may be observed (51). We propose to consider the mechanism of development of IR as a vicious circle (**Figure 1B**), which is triggered by any causes that contributes to a chronic increase in insulin release (overeating, preference for high insulin index foods such as sweets, baked goods, etc.). Excess insulin secretion stimulates overproduction of ANGII in circulating and local RASs and overgrowth of adipose tissue, which in response to insulin increases synthesis and release of ANGII into the bloodstream, further increasing its concentration (25). Since ANGII can inhibit insulin signaling, its ever-increasing levels contribute to IR. Under conditions of IR, insulin inadequately regulates glucose homeostasis and other metabolic processes due to disruption of the IRS/PI3K signaling pathway, whereas MAPK-pathway signaling is preserved, which contributes to the maintenance or even enhancement of other, non-metabolic effects of insulin (5). In these conditions insulin can affect tissues that are not directly involved in metabolism and retain insulin reactivity, thereby causing specific responses with potentially dangerous consequences including arterial hypertension (5). Because insulin promotes activation of predominantly prohypertensive components of the RAS, hypertension in patients with IR may be largely related to hyperactivation of the RAS due to compensatory hyperinsulinemia accompanying IR. Further activation of the ACE/ANGII/AT1R axis under these conditions, especially in the vascular endothelium, aggravates hypertension characteristic of obesity and IR. Excess ANGII levels in blood and tissues in IR promote profibrotic, inflammatory and hypertrophic processes causing remodeling and dysfunction of cardiovascular and renal tissues. On the other hand, the state of IR is characterized by chronic vasoconstriction in the area of insulin secretion due to high expression of AT1Rs of islet RAS on the background of high levels of ANGII, which dramatically reduces islet blood flow and negatively affects insulin synthesis and release, leading to glucose intolerance (22, 52). Further aggravation of disturbed insulin signaling in the presence of increasing ANGII, eventually leads to metabolic syndrome and T2D (53). We believe that our results may illuminate the relationship between T2D, cardiovascular disease and renal dysfunction, whose molecular mechanisms include among others IR and hyperactivity of the RAS (54).

Each of the angiotensin peptides can cause different, even opposite effects - for example, the specific action of ANGII is determined by the type of receptor it interacts with - AT1R or AT2R (17). ANGIV and ANG-(1-7), which have their own receptors, can also be AT1R and/or AT2R agonists and, depending on concentration, exert both AT1R-mediated vasoconstrictor and AT2R-mediated vasodilator effects (55–57). Insulin, by influencing the expression and function of various angiotensin receptors, may be a factor regulating their effective concentration and availability for ligands, which ultimately determines the spectrum of physiologic effects of various angiotensin peptides and maintains a physiologically adequate balance between the “pathophysiologic” and “protective” branches of the RAS.

Most hormones, including insulin, have a window of optimal physiological concentrations (58). Secretory deficiency or excess of insulin, as well as impaired signaling leading to insulin dysfunction, can disrupt the balance of regulatory interactions between insulin and RAS. When drugs affecting insulin secretion and signal transduction (insulin secretagogues and sensitizers) or insulin therapy are used, the functional activity of the RAS may shift towards the “pathophysiological” axis (59–62). This may be the cause of increased blood pressure during experimental sustained hyperinsulinemia (63) as well as the development of hypertension in pregnant women during insulin therapy for gestational diabetes mellitus (64). Various complications such as angiopathy, nephropathy, retinopathy, neuropathy, inflammation, etc., characteristic of the diabetic state (65–67) may in fact result from RAS dysregulation against insulin dysfunction due to pathology and/or medication.

In our opinion, insulin is directly involved in the flexible regulation of RAS by influencing the expression and activity of its enzymes, effector peptides and receptors. Impaired insulin signaling can lead to dysregulation of RAS function resulting in serious complications characteristic of the diabetic state. We hypothesize that IR is a consequence of RAS hyperactivation, which is provoked by excessive insulin secretion promoted by poor diet and/or other causes. Normalization of insulin secretion, primarily through dietary correction, as well as pharmacotherapy that restores the functional balance of insulin and renin-angiotensin signaling systems seem essential for the prevention and treatment of IR, hypertension and diabetic complications.

## Author contributions

TZ: Conceptualization, Validation, Writing – original draft. ST: Supervision, Writing – original draft, Conceptualization. AK: Data curation, Supervision, Validation, Writing – review & editing.
